# Sign language teaching protocol with an interactive robotic arm and real-time vision feedback

**DOI:** 10.1371/journal.pone.0345934

**Published:** 2026-10-28

**Authors:** Mireya Zapata, Andrès Rubio Proaño, Hugo Arias-Flores, Alejandro Camino, Carlos Ramos-Galarza, Jorge Banet

**Affiliations:** 1 Facultad de Ingenierías, Centro de Investigación en Mecatrónica y Sistemas Interactivos (MIST), Universidad Tecnológica Indoamérica, Quito, Ecuador; 2 Centro de Investigación de Ciencias Humanas y de la Educación (CICHE), Universidad Tecnológica Indoamérica, Quito, Ecuador; 3 Facultad de Salud y Bienestar, Carrera de Psicología, Pontificia Universidad Católica del Ecuador, Quito, Ecuador; 4 Centro de Traducciones de la PUCE, Pontificia Universidad Católica del Ecuador, Quito, Ecuador; Lamar University, UNITED STATES OF AMERICA

## Abstract

**Background:**

Sign Language (SL) is essential for Deaf communities, yet limited SL proficiency sustains communication barriers in education and services. We propose an interactive tutoring system for Ecuadorian Sign Language (EcSL) that combines real-time, vision-based gesture assessment with an embodied “reward” delivered by a robotic arm, using fruit vocabulary as an initial learning domain.

**Methods:**

We will conduct a two-arm randomized controlled experimental design, pretest–posttest control-group study with individual random assignment. About 70 university students without formal EcSL training will learn EcSL fruit signs through video and sign recognition feedback module. The system uses a webcam, MediaPipe landmark extraction, and a landmark-based fusion classifier that combines a local bidirectional LSTM with a transferred ST-GCN motion encoder; modules communicate via Robot Operating System (ROS). Outcomes. A total of six dependent variables will be assessed in both the experimental and control groups: (a) handshape, (b) palm orientation, (c) location, (d) movement, (e) non-manual signals and expressions, and (f) global coordination. Analyses will use descriptive statistics, reliability checks when applicable, and a mixed repeated-measures MANOVA.

**Discussion:**

By coupling immediate feedback with embodied reinforcement, delivered through a robotic arm, the platform aims to support early sign accuracy and provide a motivating practice environment for learners. The robotic arm responds to participants’ performance with physical rewards, thereby reinforcing correct execution through tangible, real-time stimuli. Results will guide improvements in robustness and inform extensions to broader vocabularies and deployment in educational contexts.

## Introduction

Sign Language (SL) constitutes a natural and primary linguistic medium for the Deaf and hard-of-hearing communities globally, serving as a foundation for communication, social engagement, and cultural identity [1,2]. Given its fundamental role, the scale of the community that relies on SL is both substantial and expanding; the World Health Organization projects that by 2050, nearly 2.5 billion people will experience some degree of hearing loss [3].

Despite this critical function and the growing population it serves, a widespread lack of proficiency in any specific SL among the general population and within institutions creates profound communication barriers. These barriers restrict equitable access to essential services, including education, healthcare, and legal assistance, ultimately curtailing the full exercise of fundamental human rights [4–6] and frequently leading to social exclusion [7,8].

Furthermore, this linguistic landscape is profoundly diverse, with approximately 70 million individuals currently using over 300 distinct national sign languages [9]. This plurality, evidence of the development of SLs within specific cultural contexts, presents a fundamental challenge: it fragments the user base and complicates the creation of universal technological and educational solutions.

The prevailing paradigm in communication technology design, however, has historically privileged spoken and written language, leading to architectures and standards that systematically marginalize the development of robust technological support for SL [10]. While human interpretation services remain essential, they are often constrained by limited availability, high cost, and logistical dependence. In the context of an increasingly digital society, there is therefore a pressing need to develop intelligent assistive systems capable of providing accessible, accurate, and real-time solutions to mediate communication between Deaf and hearing individuals [11,12].

To address this gap, real-time SL recognition technologies have emerged as a promising class of assistive solutions. The development of such systems is inherently challenging due to the complex visuo-kinematic nature of SL. Unlike spoken languages, which rely primarily on auditory perception, SL is a highly articulated visual language defined by five parameters: hand shape, palm orientation, movement, location, and non-manual features (e.g., facial expressions and body posture) [13–15]. Accurate signing requires the execution of all these manual and non-manual components.

Furthermore, learning and practicing SL independently face barriers such as finger self-occlusion, visual similarity among complex gestures (a primary cause of low recognition accuracy), and high variability across different signers, making automated recognition challenging in computer vision (CV). Similarly, although modern monocular RGB pipelines like MediaPipe can infer landmarks via a detector followed by a landmark regression model, the resulting landmark streams remain sensitive to self-occlusion, viewpoint changes, and motion artefacts, motivating temporal sequence modelling like Long-Short Term Memory (LSTMs) models for more robust sign recognition [10]. Consequently, the significant technical challenges, particularly in achieving reliable recognition, translate into practical barriers to the development of effective educational tools. This renders the creation of accurate, real-time, and accessible feedback systems for learners a non-trivial undertaking.

These challenges are particularly acute in educational contexts, where inclusive pedagogy aims to eliminate barriers and guarantee equitable learning opportunities for all students [16]. Inclusive education prepares students for contemporary society by fostering environments where individuals, regardless of their abilities, can learn and coexist on equal terms [17,18]. From this perspective, it becomes essential to orient an innovative paradigm that reinterprets the teaching of traditional content through flexible, student-centered methodological approaches. This paradigm fosters the exploration of new teaching approaches by combining active learning strategies and interactive technological resources that stimulate critical thinking and creativity [19–21].

A multimodal approach to learning is especially relevant, as it employs diverse representational modes (visual, auditory, kinesthetic, verbal, and symbolic) to cater to varied cognitive processing styles and deepen understanding [22,23]. For Deaf learners whose native language is visuospatial, multimodal tools such as simulators, educational videos, automatic sign language translation systems, or augmented reality environments promote inclusion by offering multiple pathways to access information [16,17]. Nevertheless, a persistent shortage of instructional materials designed for SL-native users remains a significant obstacle to knowledge acquisition.

The systemic lack of tools and interfaces that leverage visual-spatial processing for Sign Language interaction creates significant barriers to communication access and skill development for Deaf individuals. This limitation is particularly consequential in educational settings, where the absence of native-language instructional materials impedes knowledge acquisition. To address these challenges, integrating intelligent systems, specifically Computer Vision (CV) and Robotics, offers a transformative pathway to supply a positive, motivating stimulus for students. Beyond their core technical function of automating SL interpretation through the recognition mechanism, these technologies can deliver a structured, interactive, and motivating feedback mechanism. By enabling real-time performance assessment and facilitating engaging, practice-oriented interaction, CV and robotic systems can create dynamic environments that directly support and enhance the acquisition and SL proficiency.

This work directly addresses this gap by proposing the integration of intelligent systems, specifically computer vision and robotics, into an educational intervention for SL learning. Beyond automating gesture recognition, these technologies can deliver a powerful, motivating stimulus for learners. We present a combined real-time vision-based feedback and embodied robotic reward system designed to teach fruit vocabulary in Ecuadorian Sign Language (EcSL), creating a dynamic and engaging practice environment. The system provides real-time, vision-based gesture feedback. By offering interactive and immediate correction, this approach aims to directly promote and enhance the acquisition of sign language skills, contributing a novel tool to the landscape of inclusive educational technology.

## Related work

Several systems operationalise sign tutoring as a closed-loop pipeline: a camera-based recogniser evaluates the learner’s gesture and triggers corrective feedback. Piascik [24] presents an ASL fingerspelling demonstrator built around a 3D-printed forearm with multiple servos and a CNN-based classifier; OpenCV and MediaPipe are used to monitor the learner’s attempt and provide positive/negative feedback in real time. In a similar spirit, Nedjar and M’hamedi [25] introduce an interactive framework for Arabic sign letters where an AI recogniser identifies the performed sign and the robotic arm simulates the corresponding output, enabling rapid verification and repetition. These efforts emphasise the engineering requirements of low-latency inference, robust hand tracking under varying backgrounds and illumination, and a feedback policy that maps recognition confidence and error modes to actionable prompts.

Beyond recognition, sign tutoring demands accurate *sign production* on a physical platform. Uluer *et al.* [26] demonstrate a humanoid tutoring setup with a five-fingered hand and additional wrist degrees of freedom, coupled with RGB-D sensing to recognise the learner’s signs and respond via multimodal feedback. For the Ecuadorian context, Acuña-Coello *et al.* [27] present a humanoid interpreter designed for basic sign language learning using a 22-DoF torso and an interactive interface, highlighting practical integration issues such as kinematic design, actuator placement, and repeatable motion playback for sign demonstration. Collectively, these works illustrate that the fidelity of hand articulation and wrist orientation is critical for intelligible sign reproduction, and that interface design must expose sign selection, playback speed, and repetition controls without adding interaction overhead.

A key technical challenge is translating human sign datasets into feasible robot motions while preserving linguistic distinguishability. In [28], SignBot addresses this challenge through a dedicated motion retargeting stage that maps human sign sequences into robot-compatible kinematics, followed by learning-based motion control for tracking sign trajectories and a generative interaction module for sign-mediated dialogue. This line of work is particularly relevant to robotic arms with limited dexterity: retargeting and control must account for joint limits, underactuation, and the trade-off between motion smoothness and sign discriminability.

More recent tutoring architectures leverage commercially available social robots with integrated sensing and interaction primitives. As presented in [29,30], the SignPepper series proposes a Pepper-based sign language teaching assistant that combines spoken and signed interaction, and reports machine-learning-driven lesson feedback and evaluation loops. While our platform focuses on a robotic arm rather than a full humanoid, these systems inform the design of the perception–feedback loop, including multimodal cueing (e.g., verbal hints alongside gesture correction), model update strategies, and deployment considerations for real-world learning environments.

## Methods

### Research design

The research design to be implemented consists of an experimental investigation with probabilistic random assignment of participants to either the experimental or control group. All participants will complete both a pre-test and a post-test, which will be administered by an EcSL instructor who is independent of the intervention. The pre-test will assess participants’ baseline knowledge of the EcSL fruit vocabulary before any instructional activities, whereas the post-test will evaluate vocabulary acquisition following the intervention using the same standardized assessment criteria.

Participants assigned to the experimental group will receive instruction in EcSL fruit vocabulary using the proposed vision-based feedback and embodied robotic reward system. In contrast, participants assigned to the control group will not receive any instructional intervention during the study period, thereby providing a baseline for comparison of learning outcomes with the experimental group.

The study will follow a 2×2 experimental design, in which the between-subjects factor is the group assignment (experimental vs. control), and the within-subjects factor is the assessment time (pre-test vs. post-test). The intervention implemented in the experimental group consists of three instructional sessions, which are described in detail in the Detailed Procedure section. An overview of the complete experimental flow, including the pre-test, intervention sessions, control group pathway, and post-test, is presented in Fig 1.

**Fig 1:**
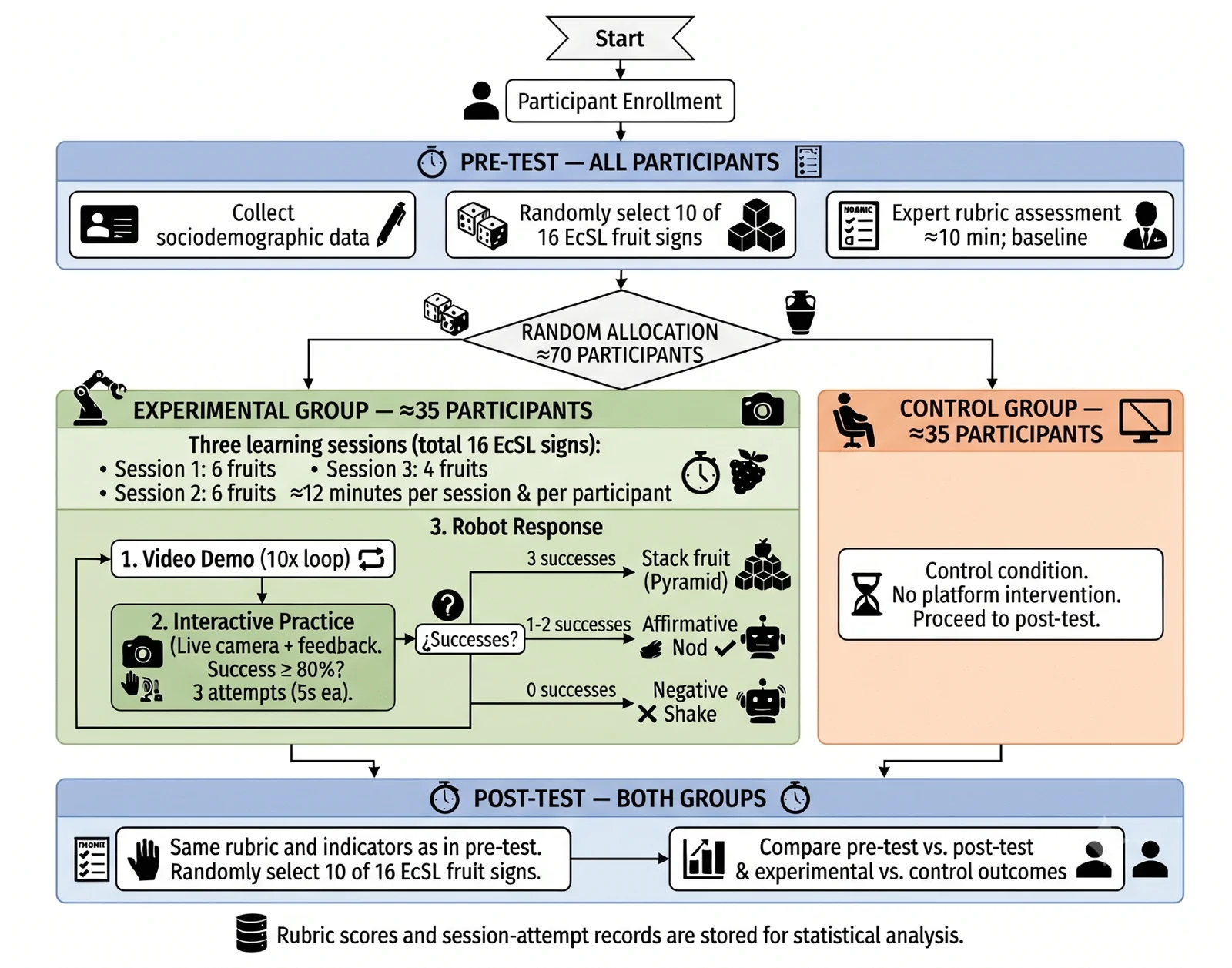
Overview of the experimental procedure. It includes pre-test, experimental group sessions, control group flow, and post-test assessment.

We expect recruitment of participants to begin on November 1st, 2026, and end on November 30th, 2026; data collection to conclude by December 2026; and preliminary results to be available by January 2027.

It is important to highlight that the individual in this manuscript has given written informed consent (as outlined in PLOS consent form) to publish these case details.

This study has been approved by the Institutional Review Board (CIRA) of Universidad Tecnológica Indoamérica (Approval No. 0034-CIRA-UTI-2026, July 10, 2026). The protocol complies with the Declaration of Helsinki, the Belmont Report, and applicable national regulations for research involving human participants. Participant recruitment has not yet commenced. All participants will provide written informed consent before enrollment. Participation will be voluntary, and participants may withdraw at any time without penalty. Images captured during the intervention will be processed only for real-time sign recognition and will not be stored. Only anonymized performance data will be retained and securely stored for research purposes in accordance with the approved protocol. Participants who withdraw may request deletion of their identifiable data before anonymization.

Results will be disseminated through peer-reviewed journals, conference presentations, and workshops. Summaries will also be shared with participating universities and academic communities.

### Setting

The experimental study will be conducted in the controlled environment of the Laboratory of the Research Center in Mechatronics and Interactive Systems at Universidad Tecnológica Indoamérica in Quito, Ecuador. The laboratory provides a standardized experimental setup, characterized by consistent ambient lighting to ensure reliable operation of the vision-based system, and ample, safe space for both robotic arm operation and participant interaction.

University students will be voluntarily invited to participate through official institutional announcements, including classroom invitations, departmental mailing lists, and campus posters. All eligible participants who provide informed consent will be enrolled in the study and will participate in both the pre-test and post-test assessments. Following the pre-test, participants will be assigned to either the experimental or control group according to the study protocol. All experimental sessions will be conducted in the laboratory under standardized conditions, where the intervention and all data collection procedures will take place., minimizes external distractions, and provides the necessary technical infrastructure for the real-time vision-based recognition system and the robotic arm used in the instructional intervention.

Participant selection will be guided by the inclusion and exclusion criteria detailed in Table 1. These criteria are designed to ensure a homogeneous participant cohort, thereby safeguarding the internal validity of the experimental outcomes and upholding ethical standards for the university students involved.

**Table 1:** Selection and exclusion criteria for study participants.

	Criterion	Description
**Inclusion**	**Age range**	Adults, 18 years or older.
	**Knowledge basis**	Participants must be enrolled in college and have no formal training in EcSL or any signed language.
	**Without disabilityor corrected/treated disabilities**	Participants without disabilities, or whose disabilities have been corrected/treated such that they do not impede the execution of the experiment.
	**Informed concent**	Participant must sign the written consent form.
**Exclusion**	**EcSL familiarity**	Have previously studied EcSL or other sign languages (defined as more than 10 hours of documented instruction). Shows familiarity with more than 50% of the target EcSL fruit signs during baseline assessment.
	**Motor limitations**	Have significant motor limitations affecting upper-limb movement necessary for signing.
	**Neurological/cognitive conditions**	Present neurological or cognitive conditions that hinder comprehension of instructions, self-reported or documented.
	**Sensory impairments**	Blind, Deaf or hard of hearing participants who are not using corrective devices, when applicable.
	**Refusal or withdrawal of consent**	Anyone who declines or withdraws consent cannot participate.

Participants will be randomly assigned in a 1:1 ratio using a computer-generated random allocation sequence created with the randomization function of R software. Allocation will be concealed using sequentially numbered opaque sealed envelopes prepared by a researcher not involved in participant recruitment or outcome assessment. Participants will be enrolled by the principal investigator after eligibility verification and informed consent. Group assignment will then be performed by an independent researcher according to the concealed allocation sequence. The EcSL instructor responsible for the pre-test and post-test assessments will remain blinded to group allocation.

### Sample size

The target population consists of university students with no formal background in SL, enrolled in undergraduate or postgraduate programs in psychology, engineering, education, health sciences, or related fields. This population was chosen because SL knowledge is increasingly relevant in inclusive educational practices, healthcare communication, and human–computer interaction contexts.

The sample size calculation was performed using G*Power software (version 3.19.7), considering a medium-to-small effect size with an Eta squared ($\eta^{2}$) value of .15, a probability of type I error (alpha) of .05, a statistical power of .90, two comparison groups, and the six dependent variables to be examined in the study. This calculation yielded a minimum required sample of 70 university students, divided into 35 students for the experimental group and 35 students for the control group.

### Outcome measures

The outcome variables used to evaluate the effectiveness of the proposed protocol are summarized in Table 2. These performance-based variables were selected to provide a comprehensive assessment of the system by evaluating the phonological parameters of EcSL. Each variable is assessed using standardized criteria that examine the correctness, consistency, temporal synchronization, and fluency of sign production. Overall, these variables provide a multidimensional evaluation of participants’ signing performance, enabling a thorough assessment of the effectiveness of the proposed teaching protocol.

**Table 2:** Outcome variables based on phonological parameters and operational definitions.

Sign parameter	Description	Evaluation criteria
Handshape	The shape of the fingers and palm during the sign execution. It includes: flexion, extension, abduction, and position of the thumb.	Conformity with the standard sign configuration Correct use of selected fingers Stability during performance Appropriate finger joint configuration (extended/bent) and thumb position consistency.
Palm orientation	The palm orientation relative to the signer’s body (up, down, toward the listener, etc.).	Correct direction of the palm Consistency throughout the sign Correct orientation according to the lexical form of the sign
Location	It is the part of the body or the space where the sign is performed (face, chest, neutral space, etc.).	Precise placement within the designated space Consistency with the standard sign Accuracy in contact or proximity
Movement	This refers to hand movement: type (straight, circular), direction, and repetition.	Proper type of movement Correct direction Rhythm and range of motion Movement timing/tempo
Non-Manual Signals – Expressions	These include facial expressions, head movements, eye contact, and body posture.	Consistency of facial expression Synchronization with the sign Required non-manual marker for the lexical or grammatical function of the sign
Global coordination	This parameter evaluates how well all phonological parameters are integrated simultaneously, rather than evaluating each one independently.	Simultaneous coordination of manual and non-manual parameters Temporal synchronization of all sign components Smooth transitions between sign components Overall fluency and naturalness of sign production

Three scoring levels will be obtained for each of them: 0 points when the sign is performed incorrectly, 1 point when it is performed moderately well, and 2 points when the participant performs the sign adequately. These scores will be assigned by a sign language expert, who will conduct the pre-test and post-test assessments in both experimental groups. The complete evaluation rubric used for these assessments is provided in Supporting Information in S1 File.

To ensure standardized outcome assessment, each EcSL sign will be evaluated using an analytic scoring rubric based on the six phonological parameters described in Table 2. For each parameter (handshape, palm orientation, location, movement, non-manual signals, and global coordination), performance will be rated on a three-point ordinal scale (0 = incorrect performance, 1 = partially correct performance, 2 = correct performance) according to predefined criteria established by an EcSL expert. A sign execution error is operationally defined as any deviation from the canonical EcSL production in one or more of these phonological parameters, including incorrect handshape configuration, palm orientation, signing location, movement trajectory or direction, omission or inappropriate use of non-manual markers, or inadequate temporal coordination among manual and non-manual components. The unit of analysis will be the individual sign performed during the pre-test and post-test assessments. Scores will be assigned independently for each target fruit sign and subsequently averaged across all evaluated signs to obtain participant-level scores for each phonological parameter. Because these outcome measures are based on ordinal expert ratings rather than raw counts of errors, the statistical analyses were selected accordingly.

### Hypotheses

1. **H1:** The experimental group will exhibit a higher level of handshape score compared to the control group when their scores are compared across pre-test and post-test measurements.
2. **H2:** The palm orientation score will be higher in the experimental group compared to the control group in the pre-test and post-test measurements.
3. **H3:** The location score will be higher in the experimental group after receiving the intervention when compared to the control group.
4. **H4:** The experimental group will exhibit a higher level of movement compared to the control group after the pre-test and post-test measurements.
5. **H5:** The experimental group will have a higher level of non-manual signals/expressions compared to the control group after the pre-test and post-test measurements.
6. **H6:** The level of global coordination will be higher in the experimental group after receiving the technological intervention when compared to the control group following the pre-test and post-test measurements.

### Materials and equipment

This section outlines the primary components of the proposed system. The diagram shown in Fig 2 introduces the components of the implemented system, outlining each one for more comprehensive visualization.

- **Edge AI Processor:** It is the computer that runs the visual interface and the sign recognition system. It also manages the communication and control of the robotic arm. The reproducible edge-inference configuration targets a Jetson Orin NX 16 GB with JetPack 5.1.3, Ubuntu 20.04 and Python 3.8, and is developed on Python For the visual interface development, the system utilizes the PyQt framework. Furthermore, the system employs the Robot Operating System (ROS), specifically ROS Noetic. It is an open platform of libraries and utilities aimed at developing robotic solutions by providing tools to manage sensors, create controllers, and transmit data [31]. An advantage of using ROS is the ability to have different code routines running in parallel and able to communicate with each other, these are called nodes [31].
- **Robotic arm:** The robotic platform used is the “myCobot 280,” with six degrees of freedom. It can work around a radius of 280 mm around its base and can manage a payload of up to 250 g, which is enough for the intended purposes of the experiment. The gripper allows the arm to pick up objects of up to 5 cm wide [32]. A RGB camera mounted on top of the gripper provides an image of 640×480 resolution, which, for the intended use in the experiment provides adequate resolution to perform accurate sign recognition.
- **Board and fruit blocks:** In order to create visually engaging and interactive materials for robot interaction, a board and a set of blocks were fabricated using 3D printing. The blocks consist of 4×4×4 cm cubes, each displaying one of the 16 fruits in the collection. Every cube face features an image of the fruit along with its name in both Spanish and English. These blocks are meant to be picked-up by the robot when the participant successfully performs the sign for a given fruit on the learning stage. The board defines the areas where the robot and specific fruit blocks are positioned and indicates the drop-off zone in front of the robot where the blocks will be stacked as the reward element, this helps to have consistent positioning of the elements and to make sure the robot predefined trajectories can interact successfully with the blocks.

**Fig 2:**
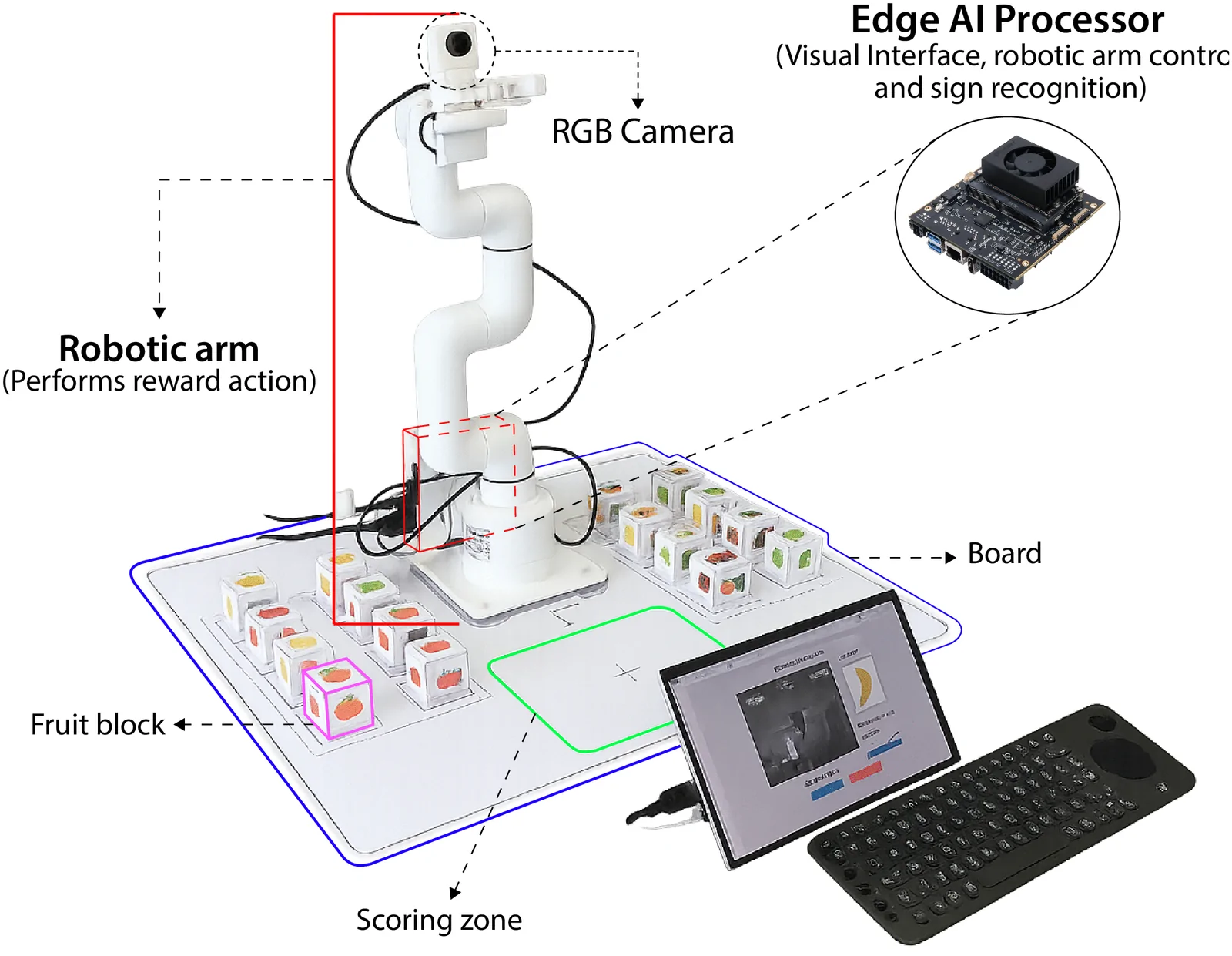
Experimental setup. In this intervention scenario, the participant is stood in front of the RGB camera.

Fig 3 shows the implemented system flowchart. As shown in the diagram, the visual interface node is the main task, while the sign recognition and robot arm control nodes run in the background. This means that the background tasks remain idle waiting for a message from the main task to start executing their logic.

**Fig 3:**
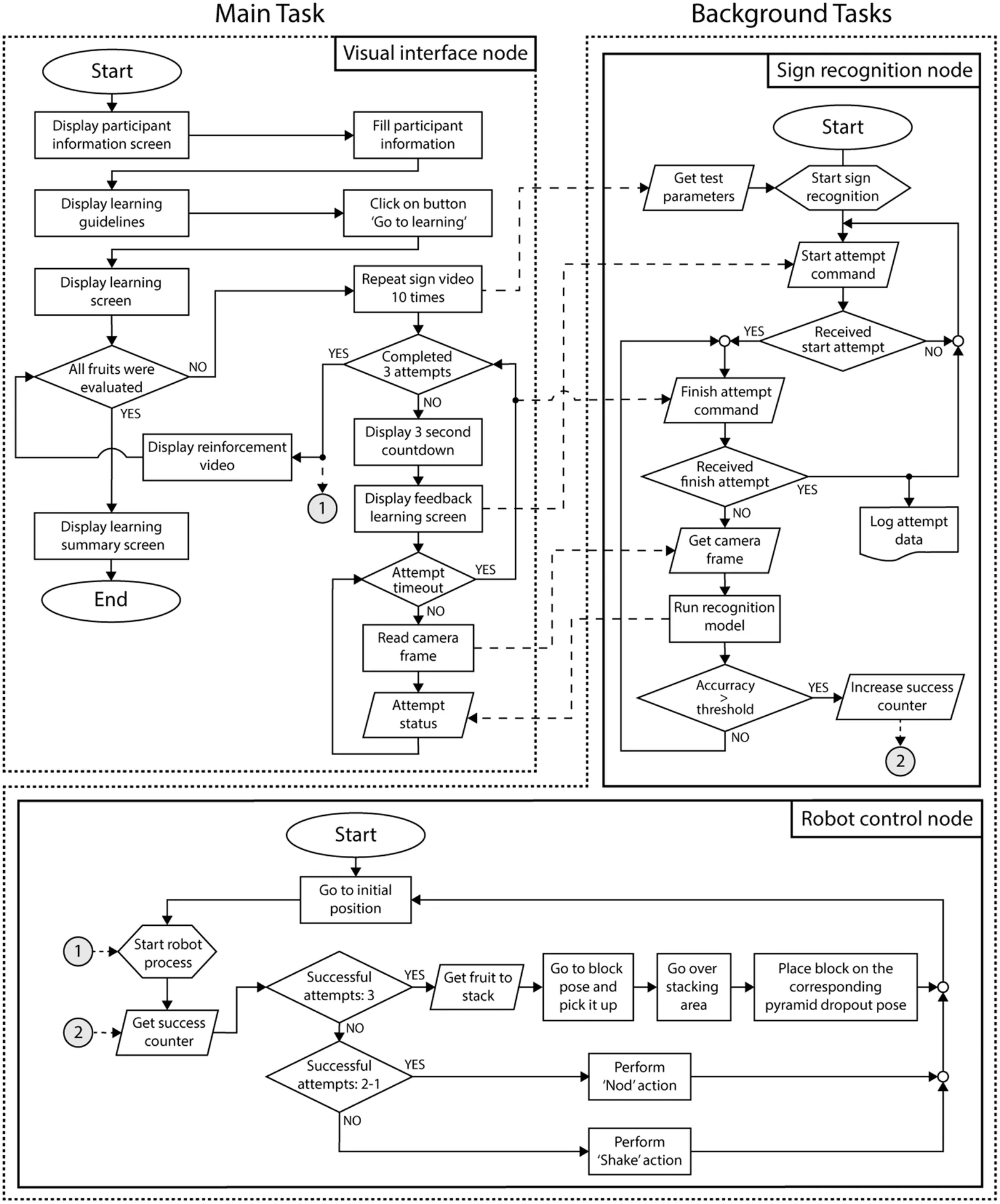
Implemented system flowchart. All the nodes are executed on the Edge AI Processor. Dashed inter-node connections represent information-carrying message pathways.

The Edge AI Processor used in the experiment includes the nodes and libraries for communicating and controlling the robotic arm. The robotic arm is connected to the Edge AI Processor via USB. Communication is carried through commands that control the robot’s joint positions and ultimately determine the robot’s movement when interacting with the system. The system is implemented using Python, integrating the visual interface developed with PyQt with the ROS framework which enables the communication between the visual interface, sign recognition and robot control nodes The Edge AI Processor runs the master node on ROS, which is the underlying node that manages communications and other nodes [31]. The main roles of each node are:

- Visual interface node: operates as the main node within a distributed architecture. In contrast, the sign recognition and robotic arm control nodes function as secondary, event-driven modules. Upon system initialization, these secondary nodes perform their requisite setup and then transition to a low-power, idle state. They persist in the background, passively awaiting specific message triggers to activate their logic. The operational structure of this node is detailed in Section Detailed procedure.

Specifically, during the evaluation phase, this master node performs two critical functions: it transmits the target fruit parameter at the commencement of each test, and it continuously streams live camera frames to the sign recognition node for real-time analysis.

- Sign recognition node: runs the model for sign recognition when it receives the target fruit and the camera frame from the messages transmitted by the visual interface node. This node outputs the sign accuracy, status, and attempt duration, among other data, to the visual interface node via a custom message type.
- Robot control node: when the model recognizes a successful attempt, it sends a signal to the robotic arm to pick up the corresponding block and place it in the drop-off zone. The robotic arm progressively stacks one block for each successfully completed task, visually representing the participant’s learning progress. Upon successful completion of all six tasks, the robot constructs a six-block pyramid at the drop-off zone located in front of it.

### Vision-based acquisition and feature extraction

The vision subsystem receives a monocular video stream from the RGB camera transmitted by the learning setup. Each frame is processed with MediaPipe Holistic, a cross-platform perception pipeline for pose, face and hand landmarks [33]. The same extractor is used offline for the training videos and online during live inference, so that the classifier sees the same representation in both settings rather than raw image pixels.

The extractor produces two synchronized representations. The local EcSL branch retains 13 upper-body pose landmarks, the 21 landmarks of each hand in both body-relative and palm-relative coordinates, and a compact set of 73 facial landmarks. Pose and global hand coordinates are centred, scaled and rotated with respect to the shoulders; local hand coordinates are normalized with the palm; and face coordinates are normalized with the eye line. The resulting static descriptor contains 510 geometric coordinates, 13 pose-visibility values and four presence masks (pose, face, left hand and right hand). It is complemented with 510 first-order motion differences, calculated only when a modality is present in consecutive frames, yielding a 1,037-dimensional input at each temporal step. Internal landmark gaps of at most three frames are interpolated; longer absences remain explicitly masked and their motion differences are set to zero.

In parallel, the transfer-learning branch preserves the unnormalized image-plane *x*,*y* coordinates of all 33 pose and 42 hand landmarks as a [*T*,75,2] sequence. This preserves the input contract of the external sign-language encoder without discarding the body-centred representation optimized for the EcSL data. Before classification, the system detects the active sign interval from robust arm, wrist and finger motion energy, retains 0.25 s of context and a minimum duration of 1 s when possible, and resamples the interval to 48 phase positions.

### EcSL fruit corpus, data augmentation, and transfer learning

The source material is the open *AI-Ready EcSL Language Corpus*, version 1.0.1, available through Zenodo under the Creative Commons Attribution 4.0 International licence (CC BY 4.0) [34]. We use a reproducible subset of 76 videos representing 16 Ecuadorian fruit signs performed by five signers. The final digit in each file name identifies the signer. During manual review, four clips marked with the suffix _x were retained in the manifest for traceability but excluded from the default experiment because their signing differed substantially from the intended form. The experiment therefore uses 72 videos. This is a selected subset of the Zenodo corpus, rather than the complete corpus.

Because each fruit has only a few examples, we create varied landmark sequences from the training videos rather than altering the images. The changes represent reasonable differences in signing timing, left–right execution, movement amplitude, camera pose, and landmark detection. The same changes are applied to both model inputs so that they remain aligned.

Table 3 summarizes the exact settings and the natural variation that each one represents. The additional views are created during training and are not stored as duplicate videos.

**Table 3:** Configuration of online landmark augmentation.

Transformation	Configuration	Variation modeled
Active window	Crop to 88–100% of the active interval	Differences in onset and offset
Temporal resampling	Phase exponent 0.80–1.25; resample to 48 positions	Signing speed and temporal alignment
Reflection	Probability 0.5, with left–right landmark exchange	Hand dominance and mirrored recordings
Local geometry	Rotation up to $\pm 7^{\circ }$, scale 0.90–1.10 and Gaussian coordinate noise (σ=0.004)	Movement amplitude, camera pose and landmark noise
Training multiplicity	Four online views per eligible video per epoch	Sample diversity without duplicate test cases
Inference	Deterministic phase views 0.90, 1.00 and 1.10; mean posterior	Stable prediction without random test-time perturbation

Signers are separated before any additional views are generated, and scaling values are calculated from training data only. Thus, neither a held-out signer’s video nor a version derived from it is used for training. During validation and live use, the system uses fixed timing views, which improves stability without treating generated views as independent test examples.

The small target set also motivates transfer learning: a model first exposed to a related sign-language task provides a useful starting point for learning the EcSL fruit signs [35]. We reuse the motion representation learned by a spatio-temporal graph convolutional network (ST-GCN) trained on ASL Citizen, a large American Sign Language (ASL) resource with 2,731 isolated sign classes [36]. This does not assume that ASL and EcSL words are equivalent: the original ASL decision layer is discarded, and only general patterns of pose and hand motion are retained. A new classifier is trained with the selected Ecuadorian videos.

To examine whether online augmentation helps, each outer leave-one-signer-out (LOSO) fold is paired with an otherwise identical run that does not use it. Both runs use the same starting point, signer-disjoint split, model, and evaluation procedure. The comparison therefore attributes observed differences to training augmentation rather than to a different held-out signer or evaluation setting.

Fig 4 presents the main error pattern and the paired comparison. Panel (a) is based on 72 out-of-fold (OOF) video predictions: each video is evaluated by a model trained without that signer’s data. Panel (b) compares macro-F1, the average F1 score across fruit classes, with and without augmentation for each held-out signer. Labels S0–S4 identify signers 0–4, and each line joins the two results for the same signer. The figure estimates the value of augmentation for this corpus and validation design; it does not claim a universal effect for EcSL. Fruit-specific recall changes are reported in supplementary tabular results.

**Fig 4:**
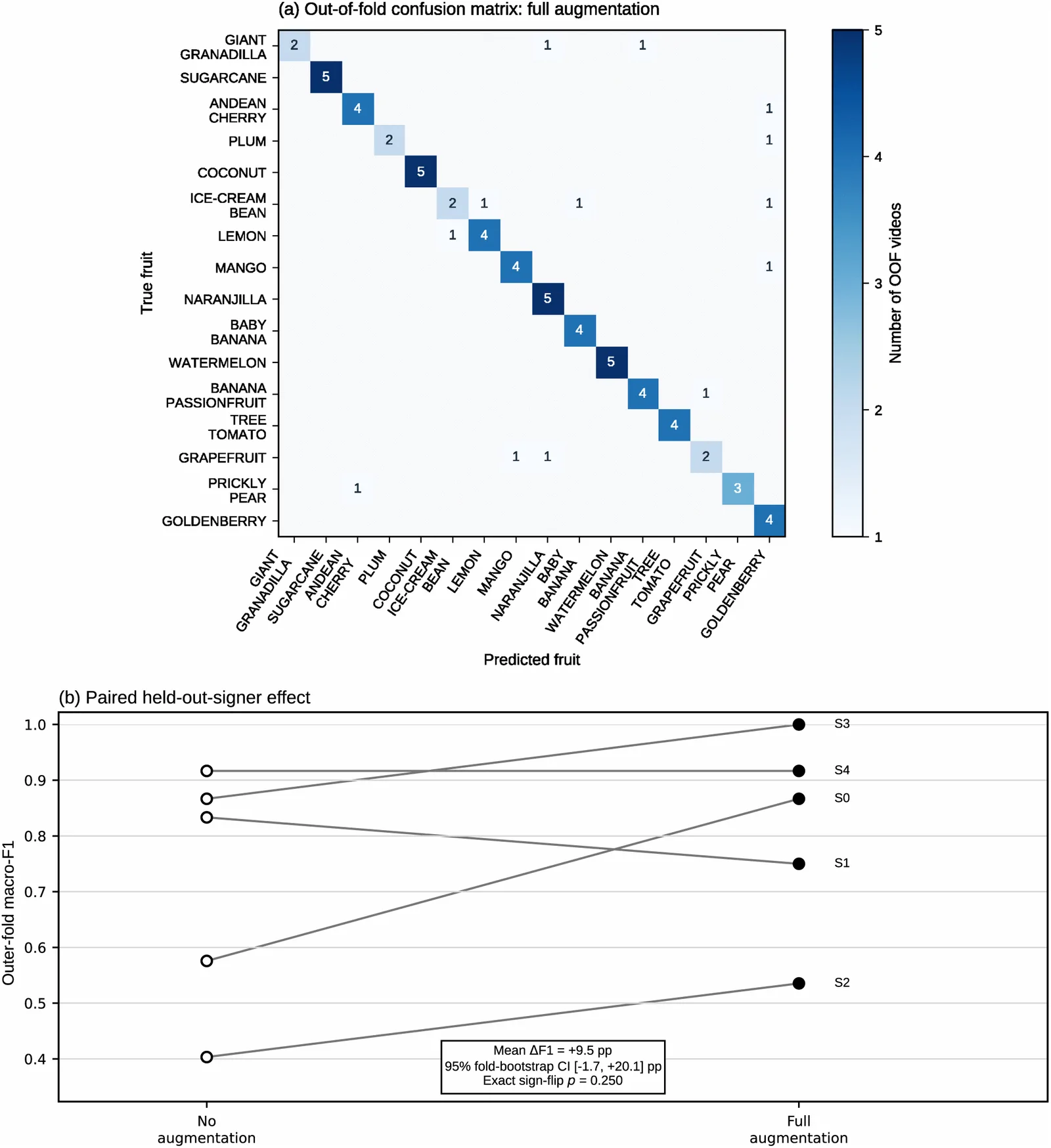
Paired estimate of the online augmentation contribution. (a) Out-of-fold (OOF) confusion matrix of the primary full-augmentation model; rows are true fruit classes, columns are predicted fruit classes, and blank cells contain zero OOF videos. (b) Macro-F1 with and without augmentation for the same five held-out signers. Labels S0–S4 identify the held-out signer, and each line joins the two results for that signer. The displayed 95% confidence interval and sign-flip result are exploratory because there are only five signers.

### Computational platform and AI model implementation

Training and deployment are deliberately separated. The complete transfer-learning experiment was trained on a Tesla V100-PCIE GPU with 16 GiB of memory, using PyTorch [37], CUDA 12.8 and automatic mixed precision in FP16. The reproducible configuration uses five outer leave-one-signer-out (LOSO) folds; within each outer-training set, four additional signer-disjoint folds choose the training epoch before refitting the outer model. This nested protocol avoids selecting the model with data from the held-out signer. The final run used 30 epochs for inner selection, a batch size of 32 and four online views per video per epoch; the final refit used the selected median epoch.

The implemented recognizer fuses two complementary branches. A compact bidirectional LSTM with temporal attention models the 48×1,037 local landmark sequence. In parallel, a ten-block ST-GCN encodes a [2,128, 27] pose-and-hand graph and produces a 256-dimensional transferred motion embedding. Both representations are projected to 96 dimensions and combined by a learned gate before the 16-class softmax classifier. To reduce overfitting on the small EcSL subset, the transferred ST-GCN is frozen during the first five epochs; from epoch six, only its final block is fine-tuned at a learning rate of 10^–5^ while its batch-normalization statistics remain fixed.

The resulting outer-fold predictions cover all 72 eligible videos and yield an out-of-fold accuracy of 81.94%, macro-precision of 84.67%, macro-recall of 81.35%, macro-F1 of 81.15% and top-three accuracy of 90.28%. These values are estimates from nested signer-disjoint validation.

For edge inference, the reproducible deployment package currently targets an NVIDIA Jetson Orin NX with 16 GB, JetPack 5.1.3, Ubuntu 20.04 and Python 3.8. OpenCV and MediaPipe Holistic extract landmarks on the CPU, while the PyTorch classifier runs locally on the Jetson GPU with CUDA and FP16. The full runtime averages five outer-fold models and one final refit model; a one-model profile is also available when lower latency is preferred, but it does not carry the above out-of-fold ensemble metric. No external server is required during use.

### Physical layout and ancillary materials

The effectiveness of the system also depends on the physical arrangement of these components in the learning space. The RGB camera is mounted on top of the gripper, ensuring a stable view of the signer. The robotic arm is positioned so that its movements are visible without requiring the learner to avert their gaze entirely from the screen. A neutral, uncluttered background is preferred to facilitate landmark detection and to avoid visual distractions.

Lighting is provided by standard LED lamps positioned to avoid strong backlighting or deep shadows on the hands and face. Although MediaPipe is robust to moderate variations in illumination, a stable and diffuse light field increases the reliability of landmark detection and improves the subjective quality of the learning experience. The workstation itself consists of an ordinary table and chair; no specialised furniture is required.

## Detailed procedure

### Data collection

As part of the registration and distribution into experimental and control group each participant’s demographic information (name, sex, and age) is collected prior to the intervention. This information is stored in a JSON file, which is subsequently loaded by the platform to identify participants and manage the intervention. The initial screen of the interface implements a drop-down menu to select the participant of the experimental group that will receive the intervention on the current session. Additionally, the interface includes another drop-down menu that allows the researcher to select one of the three predefined fruit collections corresponding to the intervention session, as described previously. Once all required data fields are completed, the *“Start learning”* button is activated, enabling the user to proceed to a guidelines screen.

### Intervention sessions

The participants will attend three experimental sessions in a laboratory setting, lasting up to twelve minutes each. According to the design of the platform, participants will learn a total of sixteen fruit-related sign language vocabulary items throughout the intervention. Accordingly, the complete set of vocabulary items will be divided into three fruit collections, with each intervention session focusing on one corresponding collection, the contents of each session are described as follows:

1. Session 1: Familiarization with the platform. During this session, the participant learns the first collection of fruits, which consists of six signs selected to be moderately easy to learn. These signs were chosen because their movements provide intuitive visual cues that facilitate the inference of the corresponding fruit. For example, the sign for *baby banana* resembles the action of peeling a banana, thereby reinforcing the association between the sign and its meaning.
2. Session 2: During this session, the participant learns the second collection of fruits, which consists of six signs selected to be of intermediate difficulty.
3. Session 3: In the final session, the participant learns the remaining four fruit signs that comprise the third collection. These signs were selected because they involve more complex movements and, therefore, are expected to present a higher level of learning difficulty.

Although every participant is presented with the same collection of fruits during the corresponding session, the order in which the fruit vocabulary is introduced is randomized. This randomization minimizes order effects, prevents specific signs from consistently benefiting or being disadvantaged by their position within the learning sequence. Consequently, any differences in learning outcomes are more likely to reflect the characteristics of the signs and the participant’s interaction with the platform rather than biases introduced by a fixed presentation order.

Based on the planned intervention activities, including instruction, sign learning, reinforcement, and robot interaction an average of one and half minutes is allocated for the learning of each fruit sign. Consequently, the first session is expected to last twelve minutes per participant, given that some extra time will be needed to get familiarized with the platform; the second session is expected to last approximately 10 minutes per participant; whereas the final session is expected to require approximately 7 minutes. During each session, participants will be positioned in front of the RGB camera associated with the robotic arm and will initiate the procedure by launching the system’s Visual Interface. The intervention is structured into three consecutive phases: Participant data, and Learning procedure

### Sign learning procedure

During this phase the participant learns each fruit of the collection in a consecutive order. The order in which the fruits are presented is randomized. The learning procedure for each fruit is divided in the following stages:

1. Video playback: The interface displays an icon representing the current fruit alongside a looping video demonstrating the corresponding sign performed by an EcSL expert signer. The video is repeated ten times, during which the participant is expected to observe and analyse the execution of the sign. No camera feedback is provided during this phase. After the reference video has been played ten times, the interface displays a three-second countdown that signals the beginning of the next phase.
2. Feedback learning: During this phase, the participant performs the sign corresponding to the current fruit. For each fruit, the participant is given three attempts, each with a fixed duration of five seconds. Throughout each attempt, the participant receives real-time feedback on the recognition accuracy of the performed sign, together with a countdown timer indicating the remaining time within the five-second attempt window. During this stage the interface shown in Fig 5 is displayed. Its components are described as follows:
  - Video playback and fruit icon: An icon representing the current fruit is displayed while the reference sign video continuously loops.
  - Camera feedback: The interface displays the live video feedback from the RGB camera mounted on the robot.
  - Attempt feedback: This section displays the sign recognition accuracy computed by the sign recognition node from the camera feed. A countdown timer is also presented, indicating the remaining time within the five-second attempt window.
  - Attempt indicators: An attempt is considered successful if the participant performs the sign within the five-second window and achieves a recognition accuracy of at least 80%. The indicator’s color denote the attempt status as follows: gray, current attempt; green, successful attempt; and red, unsuccessful attempt.
  - Learning progress indicators: These indicators summarize the learning status of each fruit within the current collection. Their color coding is as follows: gray, current fruit; green, three successful attempts; yellow, one or two successful attempts; and red, no successful attempts.
  - Attempt status message: This field displays feedback generated by the sign recognition algorithm. For example, during the attempt window, if no hands are detected within the camera frame, the participant is prompted to reposition their hands. After each attempt, the interface reports the attempt outcome together with the maximum recognition accuracy achieved during the corresponding time window.

**Fig 5:**
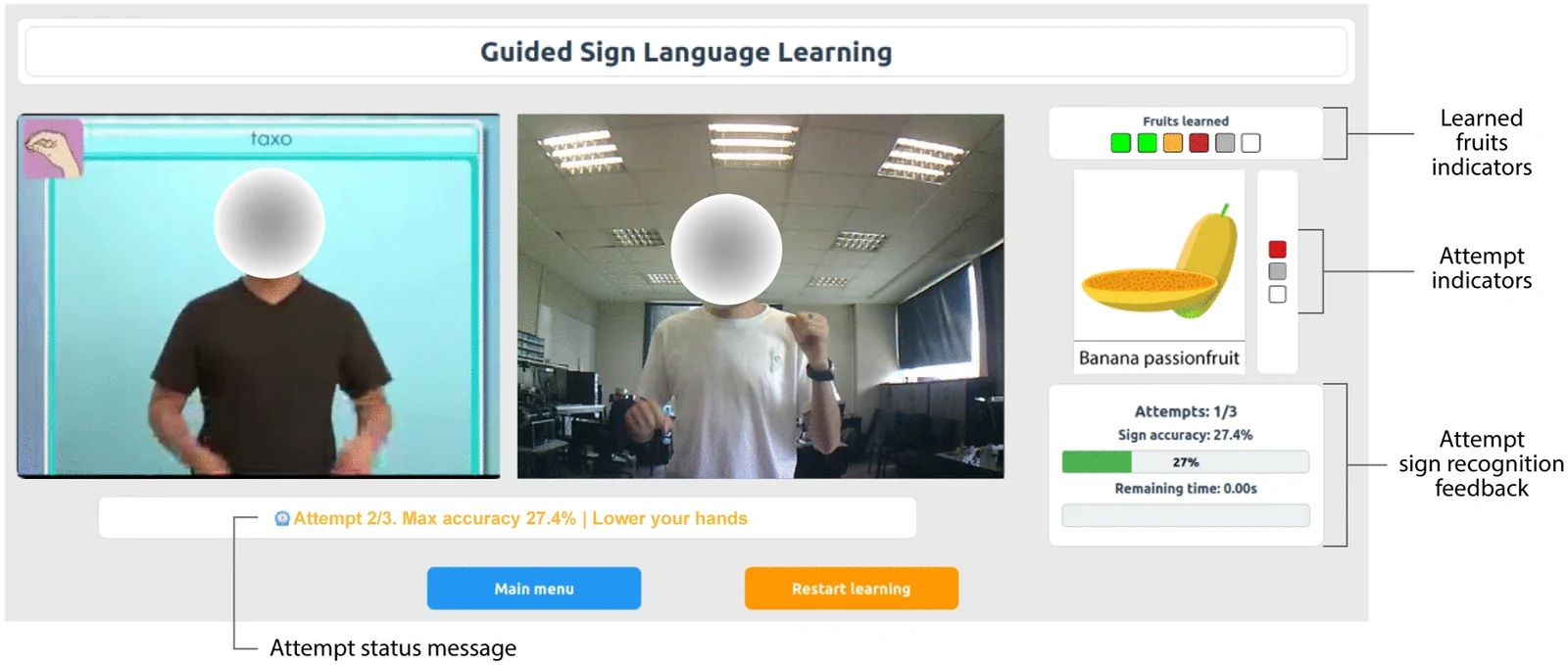
Fruit EcSL vocabulary learning screen: This screen shows a sign execution video playback on loop, live camera feedback, and sign accuracy execution as reported by the sign recognition algorithm.

After each attempt, the interface displays a three second countdown for the participant to prepare for sign execution. This cycle is repeated until all three attempts have been completed, after which the participant proceeds to the next stage.

3. Reinforcement video: After all attempts for the current fruit have been completed, the interface plays a video with subtitles in which the learned sign is used within a complete sentence. This stage aims to reinforce the sign learned during the previous phase.
4. Robotic arm interaction: During this stage, the robot performs a physical action according to the participant’s performance during the learning procedure. Once the reinforcement video concludes, the interface displays an animation of the robotic arm together with an icon representing the learning outcome. Depending on the number of successful attempts, the robot performs one of the following actions:
  - Three successful attempts: The robot moves to the location of the block corresponding to the current fruit, grasps it, and transports it to a drop-off area positioned in front of the robot, where the blocks are stacked to form a pyramid. The completed pyramid for six fruits is presented in Fig 6.
  - One or two successful attempts: The robot does not interact with the blocks but performs an affirmative nod to indicate satisfactory performance.
  - No successful attempts: The robot does not interact with the blocks and instead performs a head-shaking motion to indicate that the learning objective was not achieved.

**Fig 6:**
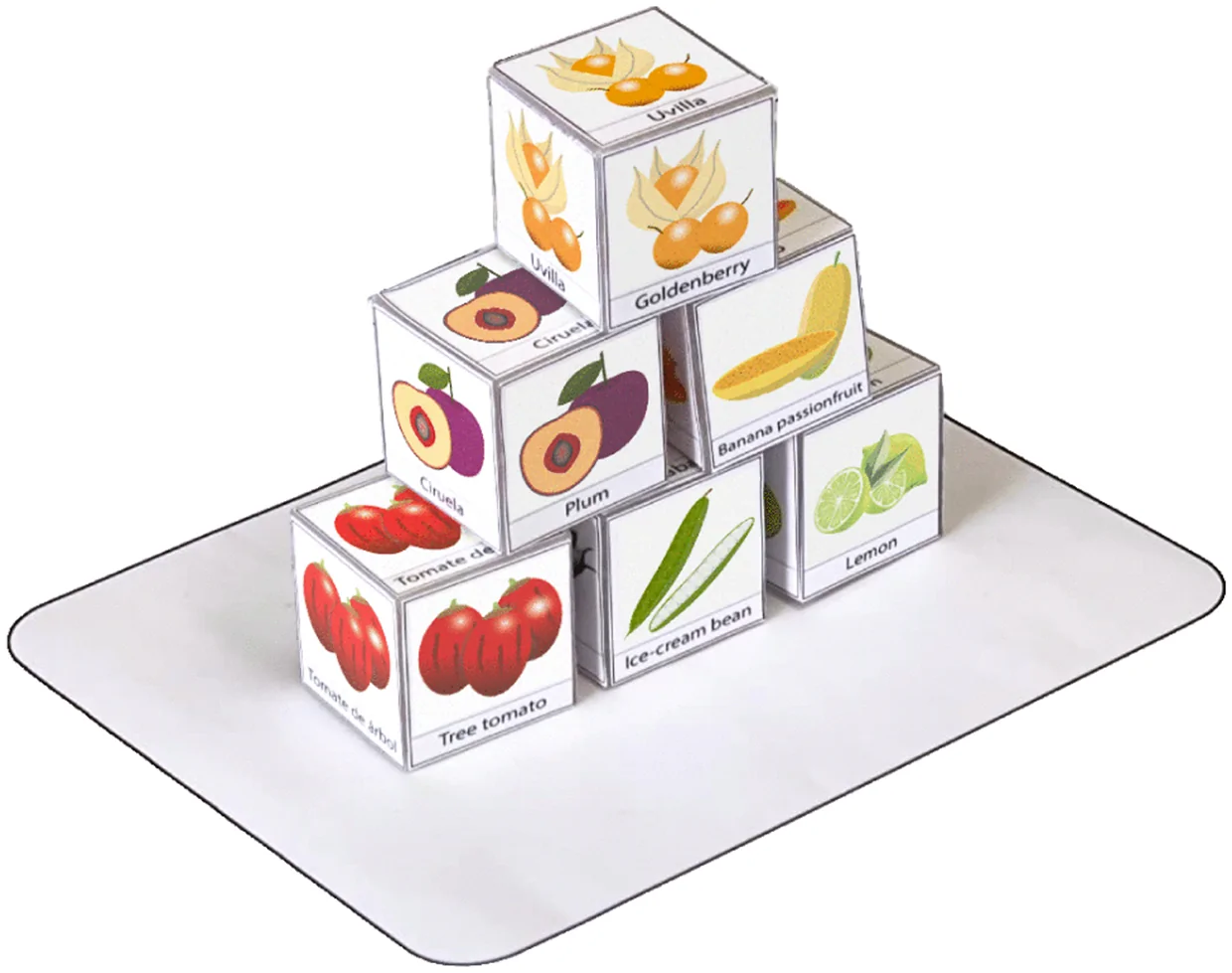
Pyramid Stack: A pyramid assembled by the robotic arm to function as a tangible reward following the correct execution of all signs within a fruit collection.

Alongside the execution of the sign recognition, following each attempt; data comprising the fruit name, the highest accuracy achieved during the attempt, its duration, and its final outcome (success or failure) are recorded in a participant-specific CSV file.

### Data analysis

Descriptive statistical procedures will be applied to summarize the data for each of the six dependent variables—Handshape, Palm Orientation, Location, Movement, Non-Manual Signals (Expressions), and Global Coordination. For each group and time point, means, standard deviations, frequencies, and percentages will be computed to provide an overview of score distributions across the three performance levels (0 = incorrect, 1 = moderately correct, 2 = correct).

Given the mixed 2×2 factorial design of this study, which includes a between-subjects factor (Group: experimental vs. control) and a within-subjects factor (Time: pretest vs. posttest), a mixed repeated-measures MANOVA will be employed as the primary analysis. This technique will allow for the simultaneous evaluation of the six dependent variables while controlling for Type I error inflation. The main outcome of interest will be the Group × Time interaction effect, which will determine whether changes in performance from pretest to posttest differ significantly between the experimental and control groups. Statistical significance will be set at α = .05, and analyses will be conducted using SPSS (version 26.0).

If the omnibus MANOVA yields a significant multivariate interaction, univariate repeated-measures ANOVAs will follow for each dependent variable, with a Bonferroni correction applied for multiple comparisons. Simple effects analyses will also be performed to decompose significant interactions. Effect sizes will be reported as partial eta squared ($\eta^{2}$p) and Cohen’s d. As a robustness check, non-parametric tests (Wilcoxon signed-rank and Mann–Whitney U) will be additionally administered to corroborate the primary findings.

### Expected results

We anticipate that the recruitment strategies will successfully yield the target sample size of 70 university students. Following randomisation, we expect the experimental and control groups to be homogenous regarding demographic covariates and to show no significant differences in baseline motor capabilities, ensuring the internal validity of the comparison.

In line with the study hypotheses, participants in the experimental group are expected to demonstrate significantly greater improvements from pre-test to post-test across all six phonological parameters of EcSL, namely handshape, palm orientation, location, movement, non-manual signals, and global coordination. These findings would indicate that the integration of real-time vision-based feedback and embodied robotic reinforcement enhances the acquisition and execution of EcSL signs compared with those achieved by participants in the control group.

In addition to what was previously mentioned, this protocol represents a beneficial advancement for Deaf and hard of hearing individuals, as learning sign language is an essential tool for communication.

With this development, individuals who are Deaf or hard of hearing will have a better option for acquiring this language. Similarly, hearing individuals can also benefit from the created protocol, as they will gain a more engaging and motivating environment for learning communication through sign language.

## Discussion

The importance of sign language for Deaf and hard of hearing individuals is widely documented in the scientific literature. Nevertheless, this population faces significant communication barriers, which limit their full access to fundamental rights such as education, healthcare, and social participation.

In this context, sign language recognition technologies emerge as an innovative solution to mediate communication between the Deaf and hard of hearing community and hearing individuals., particularly within an increasingly digitalized society. These technologies not only promote inclusion but also help develop accessible, intelligent tools that achieve communicative equity.

The proposed protocol integrates an innovative gesture recognition solution that enables real-time visualization of participants progress through positive stimuli. Specifically, the system allows users to control a robotic arm whose movement provides immediate feedback. This approach supports progressive improvement in proficiency in sign execution as difficulty increases. As a result, participants can strengthen their proficiency on EcSL, with particular emphasis on learning vocabulary related to local fruits.

Finally, the next stage of the research involves the experimental validation of the protocol through studies with human participants, taking into account the previously defined parameters. This phase will allow confirming or rejecting the proposed hypotheses and, based on the results, establishing future lines of work to further improve the developed sign language recognition system.

## Supporting information

S1 File Evaluation rubric: Item used in pretest-posttest evaluation to measure participants sign execution performance. (PDF)
