# Supplementary material for: Sign language teaching protocol with an interactive robotic arm and real-time vision feedback
Source: PLoS One. 2026 Oct 28;21(10):e0345934. doi: 10.1371/journal.pone.0345934 (PMC13716198; doi:10.1371/journal.pone.0345934)
Supplement: Supplementary file 1 [file pone.0345934.s001.pdf]

Sign Language Teaching Protocol with an Interactive Robotic Arm and Real-Time Vision Feedback

Sign Evaluation Rubric

Participant: ______________________ **Date:** ______________

Fruit evaluated:

☐ Giant Granadilla	☐ Coconut	☐ Naranjilla	☐ Tree tomato
☐ Sugar cane	☐ Icre-cream bean	☐ Baby banana	☐ Grapefruit
☐ Andean cherry	☐ Lemon	☐ Watermelon	☐ Prickly pear
☐ Plum	☐ Mango	☐ Banana passionfruit	☐ Goldenberry

Parameter	Evaluation indicator	Meets (✓/✗)	Achievement level (0–2)	Observations
**Hand configuration**	The handshape matches the standard shape of the sign			
**Palm orientation**	The orientation (vertical/horizontal and direction) is appropriate			
**Location (point of articulation)**	The sign is performed in the correct place on the body or in space			
**Movement**	The type, direction, and rhythm of the movement are correct			
**Non-manual components**	Facial and body expression consistent with the sign			
**Overall coordination**	Simultaneous integration of all parameters			

Scoring scale:

- **0 = Does not meet**
- **1 = Partially meets**
- **2 = Fully meets**
